# Plastic Debris in the Marine Environment: History and Future Challenges

**DOI:** 10.1002/gch2.201900081

**Published:** 2020-04-06

**Authors:** Imogen Ellen Napper, Richard C. Thompson

**Affiliations:** ^1^ Marine Biology and Ecology Research Centre (MBERC) School of Biological and Marine Sciences University of Plymouth Drake Circus Plymouth Devon PL4 8AA England

**Keywords:** microplastics, plastic pollution, single‐use plastics, waste management

## Abstract

The success of plastic as a material has shaped the development of modern society and challenged older materials in many of their established uses. However, plastic is now a major component of litter and is extensively reported within the marine environment. Impacts from plastic debris have been identified as a major global conservation issue with implications for maritime industries, tourism, marine life, and human health. Although there are many benefits of plastic, it is clear that society's relationship and reliance on plastics needs to be addressed. Conversely, alternative materials to replace plastic items, or solutions mitigating plastic release, also need to be critiqued to make sure their properties and environmental impacts are more beneficial. This review examines the history and impact of plastics in the marine environment. Current solutions that aim to mitigate plastics accumulation in the environment and the future challenges of plastic as a material are also discussed.

## Graphical abstract

Plastic has revolutionized modern society and challenged older materials in many of their established uses. However, plastic is now the largest component of litter and is widely reported within the environment. Society's relationship and reliance on many types of plastic needs to be addressed. This review discusses the history and impact of plastic as well as future challenges.

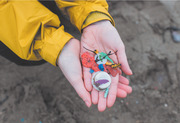

## 1 The Development of Plastic

The history of mankind is often described according to the materials used to make implements and other basic necessities. The most well‐known of these periods being the Stone Age, the Bronze Age, and the Iron Age. Currently, it could be argued that we are in the Plastic Age.^[^ ^1^ ^]^

Plastics are relatively new materials which have existed for just over a century.^[^ ^2^ ^]^ The first synthetic plastic was produced in the early 20th century. This was called “Bakelite” (a phenol formaldehyde thermoset) and it was commonly used in household items from building material to radios. However, it was not until the end of World War II that mass production of plastics began in earnest, with annual production of around 5 million tonnes in the 1950s.^[^ ^3^ ^]^

The benefits of plastics quickly became evident due to their lightweight, strong, inexpensive, durable, and corrosion‐resistant properties. Plastics are highly versatile materials which can be used to produce a range of products, from flexible to rigid items, adhesives, foams, and fibers. Consequently, the annual production of plastic increased significantly to 30 million tonnes in 1988 and then 359 million tonnes by 2018.^[^ ^3^, ^4^ ^]^

Plastic consists of synthetic or semisynthetic organic polymers. These polymers have a unique molecular structure of long chain‐like molecules made up of repeating chemical structural units. The structural units are composed of hydrocarbons which have typically been derived from fossil oil or gas feedstocks.^[^ ^5^ ^]^ There is a vast variety of different polymers, such as polyethylene, polyvinyl chloride, polystyrene, and polypropylene. Additionally, a wide variety of additives (such as fillers, plasticizers, flame retardants, thermal stabilizers, antimicrobial agents, and colorings) can also be added to enhance their performance and appearance.^[^ ^3^ ^]^

The success of plastic as a material has shaped the development of modern society and challenged older materials in many of their established uses. Numerous societal benefits are now especially evident in healthcare, agriculture, transport, construction, and packaging.^[^ ^6^ ^]^

## 2 Accumulation in the Environment

The human race generates a considerable amount of solid waste on a daily basis. Many of the plastic objects used by people every day are considered single use, convenient, and disposable. As such, plastics are a major component of waste and substantial quantities are now accumulating as litter in the marine environment.^[^ ^7^ ^]^ It is estimated that 75% of all marine litter is plastic and this debris has been reported to be accumulating on beaches,^[^ ^8^ ^]^ on shorelines of even the most remote islands,^[^ ^9^ ^]^ at the sea surface,^[^ ^10^ ^]^ in the deep sea,^[^ ^11^, ^12^ ^]^ and in arctic sea ice.^[^ ^13^ ^]^ There is also increasing awareness of the accumulation of plastic litter on land as well as in freshwater habitats.^[^ ^14^, ^15^, ^16^ ^]^

In the 1980s, roughly 30 years after the start of mass production, marine plastic debris was first identified as a potential wild scale impact to the marine environment worldwide.^[^ ^17^, ^18^ ^]^ Some of the earliest reports of plastic debris in the ocean were of small floating particles that were captured in surface‐towing plankton nets.^[^ ^19^, ^20^, ^21^ ^]^ Other reports included synthetic fibers in water samples,^[^ ^22^ ^]^ shipboard visual observations of large floating debris,^[^ ^23^ ^]^ seafloor debris in benthic fishing trawls,^[^ ^24^ ^]^ and plastic debris on beaches.^[^ ^25^, ^26^ ^]^ Although marine plastic pollution was reported decades ago, it has only recently been recognized as a pervasive global issue.^[^ ^2^, ^27^, ^28^, ^29^ ^]^

Plastic debris can be defined and described in a variety of ways. Frequent descriptors are shape (spheres, beads, pellets, foams, fibers, fragments, films, and flakes),^[^ ^30^, ^31^, ^32^ ^]^ color, polymer type, origin, and original usage (e.g., packaging).^[^ ^30^, ^33^ ^]^ For example, research by Kor and Mehdinia in 2020 evaluated plastic abundance in surface waters of the Persian Gulf and reported that majority of plastics collected were polyethylene (48%) and polypropylene (28%) and the predominant colors were blue and white. In terms of their shape, 44.1% of the collected plastic was fibers, followed by fragments (29.0%), film (14.7%), and pellets (12.2%).^[^ ^34^ ^]^ Whereas research by González‐Hernández et al. in 2020, analyzed microplastic debris in Playa Grande beach (Tenerife, Canary Islands, Spain) and reported that polypropylene and polyethylene accounted for 76% and 19% of the total, respectively. Among the plastics found, 83% were fragments, 11% pellets, 4% fibers, and 2% films.^[^ ^35^ ^]^

Size is typically the most common reporting descriptor, as this encompasses all plastic types. However, there can be ambiguous terminology for the sizes of plastic which can compromise progress in research and mitigation measures. As well as size classes, Hartmann et al. in 2019 have produced a framework which also focuses on physicochemical properties (polymer composition, solid state, solubility) as defining criteria with size, shape, color, and origin as classifiers for categorization.^[^ ^36^ ^]^

The three categories that are typically used to describe the size of plastic contamination are macroplastic (>20 mm diameter), mesoplastic (5–20 mm), and microplastic (<5 mm).^[^ ^1^, ^37^ ^]^ However, nanoplastics are more frequently being reported (<1000 nm).

### 2.1 Macroplastic

Macroplastic pollution refers to plastic items larger than 20 mm and due to its high visibility, contamination to the environment by macroplastic may be perceived as one of the most concerning forms of plastic pollution. Its accumulation has been widely reported since the 1990s and has been found in a wide range of habitats.^[^ ^29^, ^38^, ^39^, ^40^, ^41^ ^]^ Due to the size of this debris, it is often possible to categorize items according to their original usage, e.g., packaging, fishing, or sewage‐related debris.

Macroplastic can enter the environment from many sources, generally split into oceanic or land based. Oceanic sources include fishing, boating, and shipping—including gradual breakdown of rope and polymer‐based paints. Land‐based sources include primary industry, litter, sewage, and storm water.^[^ ^42^, ^43^, ^44^, ^45^ ^]^

Clean‐up campaigns typically focus on these larger items as they are more visible and normally easier to find. However, there is wide geographical variability in abundance, which increases the difficulty of analyzing potential trends.^[^ ^8^ ^]^

### 2.2 Microplastic

The presence of small plastic fragments in the open ocean was reported for the first time in the 1970s.^[^ ^19^ ^]^ However, it was not until 2004 that the term “microplastics” was coined in a paper describing the long‐term accumulation of fragments just a few microns in diameter.^[^ ^46^ ^]^ Until recently, microplastics have been a largely overlooked part of plastic pollution monitoring. Within the last decade, with accumulating data on the impact and consequences of such debris, the topic has received increasing research interest.^[^ ^27^ ^]^

There are two main classifications of microplastic: primary and secondary sources.

Primary microplastic directly enters the environment in the microplastic size (<5 mm in diameter). These are produced through extrusion or grinding, either as a feed stock for manufacture of products^[^ ^47^ ^]^ or for direct use,^[^ ^48^ ^]^ e.g., in cleaning products,^[^ ^33^, ^49^ ^]^ microbeads in cosmetics^[^ ^50^, ^51^ ^]^ and as air‐blasting media.^[^ ^52^ ^]^

Microplastics resulting from the fragmentation of other plastic items/pieces are described as secondary microplastics. Additionally, this can include microplastics from the generation of particles during product use, e.g., tire wear or fibers from clothing.^[^ ^33^, ^53^, ^54^ ^]^ It is predicted that even if emissions of larger items of plastic to the environment were to immediately stop, it is likely that we would still see an increase in the quantity of microplastic as a consequence of the fragmentation of larger items that are already in the environment.^[^ ^55^ ^]^

Microplastics greatly outnumber large plastic items in marine systems, but only account for a small proportion of the total mass of plastic in the ocean.^[^ ^56^, ^57^ ^]^ However, it is now apparent that microplastics are a ubiquitous component of anthropogenic debris in marine and freshwater environments.^[^ ^13^, ^54^, ^58^, ^59^, ^60^ ^]^

### 2.3 Nanoplastic

Nanoplastics are less than 1000 nm in size. They also can be from primary or secondary sources.^[^ ^27^ ^]^ Like microplastics, nanoplastics have been an overlooked part of plastic pollution in recent years, but now there are increasing studies on the identification of nanoplastic sources.^[^ ^27^, ^61^ ^]^ However, there is not conclusive data about their impacts.^[^ ^62^ ^]^ It is likely that all plastic breaks down into nanoplastic size before complete degradation and mineralization. Therefore, the presence of nanoplastics in the marine environment is likely to be of increasing significance in the years to come.

## 3 Degradation and Mineralization

The degradation process for plastics is ongoing and the material fragments into smaller and smaller pieces over time; eventually breaking down into microplastic, and then probably further into nanoplastic particles.^[^ ^33^ ^]^ The rate of degradation depends on the chemistry of the polymer and where it was exposed.^[^ ^27^, ^63^ ^]^

Initial degradation converts the plastic polymers into smaller, more fragmented units and introduces new chemical groups to the ends of the carbon chain, changing the nature of the compound. This process is followed by biotic degradation (mineralization), which converts the carbon atoms into carbon dioxide (CO_2_) and inorganic chemicals. However, this is a very lengthy process and further considerable degradation would be required before the plastic would reach a size where it might biodegrade completely. The timeframe for the complete mineralization of plastic is unknown, but Andrady in 2005 suggests that all of the conventional plastic ever made is still with us on the planet in a form too large to biodegrade, except if burnt.^[^ ^64^ ^]^

Fragmentation in the marine environment is dominated by physical mechanisms including weathering due to UV radiation, mechanical (abrasion, wave action, and turbulence), thermal, and chemical action.^[^ ^65^ ^]^ UV radiation in sunlight causes oxidation of the polymer matrix, resulting in chemical bond breakage. According to Corcoran et al., beaches are the optimal settings for plastic fragmentation due to the presence of both chemical and mechanical weathering.^[^ ^66^ ^]^

Rates of degradation may be reduced in the seawater due to reduced light levels, lower temperatures, and saline conditions.^[^ ^67^ ^]^ Colonization of microorganisms, plants, algae, and marine life onto floating plastic in the ocean (a process described as fouling) can also inhibit degradation.^[^ ^68^ ^]^ First, the biofilm may “shield” the plastic from UV light^[^ ^69^ ^]^ and since exposure to UV enhances degradation, fouling will likely reduce any photo‐degradation rates. Second, fouling can make plastics negatively buoyant causing buoyant items to sink^[^ ^70^ ^]^ and hence further reducing irradiance.

More research is needed to understand long‐term weathering of plastic and the relationships between weathering properties and sorption capacities toward pollutants.^[^ ^71^ ^]^

## 4 Sources

Over 359 million tonnes of plastic is produced annually and production has been predicted to double in the next 20 years.^[^ ^4^, ^72^ ^]^ More than 40% of this amount is for single‐use applications which can include plastic carrier bags, cutlery, straws, cups, and food packaging.^[^ ^2^, ^73^ ^]^ These short‐lived applications rapidly lead to large amounts of persistent plastic waste and a proportion of this waste can enter the environment as litter. It is estimated that 8 million tonnes of mismanaged plastic waste enters the oceans every year and there is evidence of increasing quantities over time.^[^ ^7^, ^74^ ^]^ The majority of this plastic originates from inland sources and is emitted to the oceans from coastlines or rivers.^[^ ^7^ ^]^ It has been predicted that rivers are a major transport pathway for marine plastics, transporting 1.15 to 2.41 million tonnes of plastic waste potentially entering the ocean per year.^[^ ^75^ ^]^

Assuming there are no improvements in waste management infrastructure, the cumulative quantity of plastic waste available to enter the marine environment from land could increase by approximately three times over the decade up to 2025.^[^ ^7^ ^]^ Microplastics have been detected at very high levels globally in rivers and lakes which could further add to this estimation.^[^ ^76^, ^77^, ^78^ ^]^ Due to the scale of the issue, plastic waste in the marine environment has been identified as a major global issue by the United Nations Environment Assembly and in the G7 Leader´s declaration 2015.^[^ ^79^, ^80^, ^81^ ^]^

Plastic can also enter the marine environment from sources that are typically not considered as generic waste. Over 7 00 000 microplastic fibers are estimated to be released from a typical 6 kg wash of synthetic clothing.^[^ ^82^ ^]^ It has also been suggested that plastic teabags might release ≈11.6 billion microplastics and 3.1 billion nanoplastics into a single cup per beverage.^[^ ^83^ ^]^ Tire wear has also been considered as a substantial microplastic emitter into the environment, where it has been estimated to contribute 28% of secondary microplastics to global oceans (4 20 000 tonnes yr^−1^).^[^ ^84^ ^]^ These various sources of microplastics may enter the marine environment by bypassing waste water treatment plants, through storm drains or even be carried in the air and deposited at sea.^[^ ^85^, ^86^ ^]^

Plastic can also be released from ocean‐based sources such as shipping and aquaculture.^[^ ^27^, ^87^ ^]^ Due to its low cost and durability, fishing gear is mostly made from plastic. Items that comprise abandoned, lost, or otherwise discarded fishing gear are extremely heterogenous in terms of polymer type, size, shape, and color. For instance, some discarded or abandoned nets can be 100s of meters in length, while offcuts are typically < 5 mm (UNEP in 2016). The structure of synthetic rope is highly durable, but its plastic material is susceptible to embrittlement, cracking, and reduction in mechanical properties. This leads to fragmentation and the formation of secondary microplastics.^[^ ^65^ ^]^ Therefore, the fragmentation could also result in the release of large quantities of microplastic particles into the marine environment.^[^ ^88^ ^]^

## 5 Distribution

Once in the marine environment, plastic can become widely transported due to its properties of buoyancy and durability. Particles of low density tend to stay in surface water and could transfer horizontally. Denser particles are more likely to transfer vertically, e.g., 5 mm polyoxymethylene particles, which have a density of 1.6 g cm^−3^, could settle through the water column of ≈250 m in the central Gotland basin in <18 h.^[^ ^89^ ^]^ Additionally, biofouling may also act as a mechanism increasing sedimentation.^[^ ^27^, ^90^ ^]^

However, quantifying the abundance and distribution of plastic is strongly influenced by the sampling method chosen and this can vary by location and debris size. At present, most methods depend on some degree of visual selection of items or particles. The most direct visual selection methods occur in surveys of debris on beaches.^[^ ^29^ ^]^ Visual selection can also occur at the sea surface from ships or aircraft, and on the seafloor by divers or towed underwater camera systems, in which only debris visible to the observer (for direct observation) or to the analyst (for photographs or video) is recorded.^[^ ^91^ ^]^

Prevailing weather may also be an important factor in distribution. Research by Prata et al. investigated the effects of seasonal factors on the characteristics of (micro)plastics in a sandy beach in Aveiro, Portugal. The results found that pellets of polyethylene were more abundant during wet seasons, while fragments and pellets of both polyethylene and polypropylene characterized dry seasons. A higher concentration of plastic fibers was also found during dry seasons, likely from their accumulation and beach use during bathing season.^[^ ^92^ ^]^

## 6 The Impact of Plastic in the Marine Environment

There are a variety of potential impacts plastic can have within the marine environment. The European Union Marine Strategy Framework Directive (MSFD) expert group on marine litter recently concluded that plastics present a “large scale and serious threat to the welfare of marine animals.”^[^ ^79^ ^]^ More than 700 species of marine organisms have been reported to encounter plastic debris, which can result in severe physical harm or death, or more subtle effects on behavior and ecological interactions (e.g., the ability to escape from predators or migrate).^[^ ^93^, ^94^ ^]^

The most visible effect of plastic pollution on marine organisms is entanglement of organisms in marine debris, often in discarded or lost fishing gear and ropes.^[^ ^95^, ^96^ ^]^ Entangled organisms can be hindered in their ability to move, feed, and breathe. In addition, many marine organisms mistake litter for food and ingest it.^[^ ^94^, ^97^, ^98^, ^99^ ^]^ Ingestion of plastics by sea turtles^[^ ^100^ ^]^ and seabirds^[^ ^101^ ^]^ was first documented in the 1960s. Recently, microplastics were reported in all digestive tracts of ten species of marine mammals stranded around the British coast.^[^ ^8^ ^]^

Laboratory studies predict uptake of even smaller particles, in the nano‐size range, may be rapid.^[^ ^102^ ^]^ For example, Al‐Sid‐Cheikh et al. found nanoplastics could accumulate in scallops (*Pecten maximus*) within 6 h of experimental exposure, but many were subsequently excreted back to the marine environment.^[^ ^102^ ^]^ Laboratory experiments have shown that ingested plastics may accumulate in the stomach of organisms and affect individual fitness, with potential consequences for reproduction and growth.^[^ ^98^, ^103^ ^]^ It was also observed that microplastics 31.5 µm in size, in the digestive systems of planktonic crustacean were alternated to <1 µm in diameter,^[^ ^104^ ^]^ suggesting that secondary nanoplastic could also be produced by fragmentation of microplastic inside the digestive organ of animals after ingestion.^[^ ^105^ ^]^

Plastics may transfer contaminants sorbed from surrounding water, such as endocrine disruptors and persistent organic pollutants.^[^ ^51^, ^106^, ^107^, ^108^ ^]^ Additive chemicals can be present in high concentrations and it is considered their release could provide an important pathway for chemical transfer to biota.^[^ ^109^, ^110^ ^]^ For example, a recent study in Korea demonstrated that potentially harmful flame retardants could be released from buoys used in an aquaculture facility, leading to elevated concentrations of flame retardants in the surrounding environment.^[^ ^111^ ^]^ However, modeling estimates indicate the amount of chemical transfer from water to organisms via plastic is probably not a major pathway leading to harm.^[^ ^112^ ^]^ Additionally, more work will be needed to establish the extent to which chemical additives incorporated in plastic items at the time of manufacture could transfer to organisms in sufficient quantities to be harmful.

The durability and buoyancy of plastics present the possibility of transporting species horizontally to ecosystems where they are not native^[^ ^113^ ^]^ or vertically from the sea surface through the water column to the seafloor.^[^ ^114^ ^]^ Microorganisms from the Vibrio family were shown to be capable of rafting on plastics and microplastics.^[^ ^115^ ^]^ Plastic may also offer habitats, e.g., the insect *Halobates micans* has been shown to use plastic litter as oviposition sites.^[^ ^116^ ^]^ Sediments have also been smothered with nonbuoyant plastic items affecting gaseous exchange and altering the composition of species present in assemblages.^[^ ^117^, ^118^ ^]^ Marine plastics may also cause more greenhouse gas emissions due to impacting ecosystems responsible for the gas exchange and circulation of marine CO_2_.^[^ ^119^ ^]^

The substantial quantities of plastics that are entering aquatic habitats daily can present a range of negative economic and environmental consequences.^[^ ^7^, ^120^ ^]^ Plastic debris can have negative economic consequences on navigation, aquaculture, tourism, and fisheries. Stranded plastic along shorelines creates an aesthetic issue, which has negative impacts for tourism.^[^ ^121^ ^]^ In terms of fisheries, plastic litter can reduce or damage catches and vessels. There is also emerging evidence that even small quantities of litter on beaches may have a negative effect on human well‐being.^[^ ^122^ ^]^

Plastic waste in the environment can have substantial economic costs associated with it. For example, Beaumont et al. estimate that the economic costs of marine plastic, as related to marine natural capital, are conservatively conjectured at between $3300 and $33 000 per tonne of marine plastic per year (based on 2011 ecosystem service values and marine plastic stocks).^[^ ^123^ ^]^ The full economic cost is likely to be far greater as this value includes only marine natural capital impacts.^[^ ^123^ ^]^

One of the main obstacles in the advance of knowledge in the consequences of plastic pollution is the absence of harmonization of assessment methodologies (sampling and analysis). There are inconsistencies among the research results because there are limits in the accuracy of the sizes and possible concentrations in the environment due to the analytical instrumentation.^[^ ^124^ ^]^ Therefore, although scientific evidence on the impact of plastic is rapidly increasing, many critical issues such as persistence and accumulation are still poorly understood. Without consensus in a standardization of analytical methods for collection, identification and quantification of plastics in all size ranges in the environment, their concentrations, spatial and temporal changes, and risks will be unclear.

## 7 Solutions and Future Challenges

There are many areas where the use of plastics can make a positive contribution to society and the environment. However, it is equally clear that some single use items are not necessary, especially when considered in the context of the associated waste generation. Littering of the aquatic environment by plastics is attributed to a number of factors. These can include human population density, increase of plastic production, improvement of living standards facilitated by so‐called disposable society and associated consumer behavior. As a consequence, the potential solutions to mitigate the problem are widespread and complex. However, changes from industry, governments, and increased consumer awareness can have a key role in helping reduce the potential for end of life plastic to become waste and litter.

### 7.1 Industry

Focusing on industry, disposal pathways for a product need to be considered right from the design stage. Long‐term sustainable solutions require moving from a linear economy toward a more circular economy.^[^ ^125^, ^126^ ^]^ Although most plastics are inherently recyclable, many single‐use items are not currently designed to be widely compatible with recycling. Waste reduction can be achieved by a combination of sustainable production and consumption patterns, and more circular use of materials, e.g., designing products which avoid unnecessary plastics usage or are made to be more re‐usable and recyclable. A key challenge therefore is to ensure end‐of‐life is appropriately considered right from the design stage. In the views of the authors, there are many examples of products where recyclability has been severely compromised by inadequate design.

It should also be noted that there is a rise of items, such as shoes and clothes, that claim to be made out of plastic waste collected from the ocean or beach. Although providing a benefit in terms of removing waste in the natural environment and education to the consumer, the environmental implications of such products remain unproven. Therefore, there should be consideration in product development so redeveloped products do not themselves become possible sources of plastic pollution again.^[^ ^45^ ^]^

### 7.2 Government Intervention

Various measures have already been undertaken at local, national, regional, and international levels.^[^ ^127^ ^]^ Governments have a key role in mitigating plastic accumulation into the environment. Systemic changes may be facilitated by policy initiatives, e.g., a tax may be required on nonrecyclable products or an incentive to use recycled content in new products so as to encourage reuse and or design for recyclability. Policy can create the essential legislative framework to stimulate mitigation actions that contribute to a reduction in plastic waste at source, as well as encouraging the clean‐up of plastic pollution on coastlines. For example, the EU has announced a ban on single‐use cutlery, cotton buds, straws, and stirrers from 2021, as well as a reduction of other plastic items that are not included in the ban by at least 25% by 2025 in each member state.^[^ ^128^ ^]^ The UK also has a plan to tax the manufacture and import of plastic packaging that contains less than 30% recycled plastic.^[^ ^129^ ^]^

However, despite existing efforts, the amount of plastic waste continues to increase and plastic leakage into rivers and oceans persists. Currently there is little consistency of regulations between countries. Understandably, there will be variations in plastic waste at a local level, but it is recommended that governments need to cooperate globally or nationally to regulate the major sources of microplastics, namely, industrial and domestic products.^[^ ^130^ ^]^

Raubenheimer and Urho have suggested the introduction of a global extended producer responsibility (EPR) scheme. The overall objective of a global EPR scheme is to provide assistance to countries in regulating the features of plastic products placed on their market based on the availability and capacity of national waste management services. This scheme could provide a tool to drive innovation as it will give a market advantage to companies that strive to meet them. Implementation could result in minimization of residual waste, making end‐of‐life plastics a valuable raw material for recycling purposes and reducing leakage of plastics into the environment.^[^ ^131^ ^]^

### 7.3 Public Awareness and Education

Litter can be defined as something of little or no value and hence the problem may be exacerbated because plastics are inexpensive, which facilitates short‐lived applications. However, it has been suggested that marine litter can be used as a vehicle to inspire and promote more sustainable economies and lifestyles.^[^ ^132^ ^]^ Education, outreach, and awareness are effective ways to address marine litter.^[^ ^133^ ^]^ This is because improving public awareness of the problems produced by plastic debris is an important step toward changing people's behavior with regard to plastic consumption.

There has been a rise in citizen science and outreach activities focusing on plastic pollution. The Marine Conservation society in the UK attracted record numbers for its annual Great British Beach Clean with 15 000 volunteers; double the number in 2017.^[^ ^134^ ^]^ This suggests that the public are becoming more engaged in the issue and wanting to take action. Additionally, citizen science projects can engage the public, while also collecting relevant data for scientists. For example, Camins et al. discuss how a lightweight and low‐cost paddle trawl towed behind paddle surfers could obtain samples for microplastic characterization and quantification nearshore for potential research purposes.^[^ ^135^ ^]^

However, there needs to be education and change in behavior right along the supply chain in order to develop long‐term sustainable solutions. This could be facilitated by greater dialog between the various stakeholders from design, through production and use, to disposal. Overall, we need greater stewardship so that the benefits of plastic can be realized without the accumulation of unnecessary waste in managed systems and in the environment.

Children can also be very influential to create change in society; not only because they represent the next generation of consumers and decision‐makers but often they can inspire and influence directly the behavior of their families and close community.^[^ ^136^ ^]^ For example, it was found that educational activities about marine litter can enhance understanding and concern surrounding it in UK school children, and therefore children have an important role to play.^[^ ^137^ ^]^

### 7.4 Waste Management

The accumulation of plastic litter in the oceans is actually a symptom of a wider, more systemic problem of the linear use of plastic materials and the rapid accumulation of waste on land. One study suggests that unless waste management improves profoundly in the coming years, by 2025 the amount of plastic waste entering the ocean from land could be three times greater than it was a decade previously.^[^ ^7^ ^]^

A range of preventive measures exist. These include recycling, banning plastics, improving port reception facilities, and incentives/disincentives relating to littering. An array of these measures are already being implemented.^[^ ^138^, ^139^, ^140^ ^]^

Waste management frameworks are typically designed to help minimize loss to the environment, but management practices can differ considerably between countries. Incorrectly managed systems may cause waste to escape into the environment. In industrialized countries, landfills are usually covered regularly with soil or a synthetic material, and the landfill is cordoned by a fence to prevent any waste accidentally leakage. However, this is often not the case in developing regions.^[^ ^7^, ^37^ ^]^ There are also circumstances in which waste management will not suffice in stopping plastic leaking into the ocean. For example, in the immediate aftermath of a tropical storm or flood, resource management is understandably focused on human health as opposed to waste management.^[^ ^141^ ^]^

Recycling can be used to increase its circularity and material flows. However, it can be expensive and is reliant on human behavioral engagement. It can also produce lower quality materials, in terms of both thermal and mechanical properties. More effective and longer‐term change to a more circular economy will require consideration of end‐of‐life recyclability from the design stage.

The variety of different plastic types also presents a complication for the viability of recycling and the quantity and diversity of single‐use products is putting increasing pressure on waste management infrastructures, e.g., separating different material types into appropriate recycling streams. However, on a global scale, a substantial proportion of the human population is not connected to waste management systems. Hence, a key focus is on improving solid waste collection and management. In the interim for countries with relatively poor waste management infrastructure, e.g., a lack of recycling plants, mandatory take back schemes could be used as a policy intervention such that there is a requirement on those exporting to such nations that they take back and responsibly deal with end‐of‐life plastic.

### 7.5 The Future of Plastic

Production of plastic is likely to rise substantially during the next few decades. With the many benefits that plastic provides, it is difficult to see modern society without it as a core material. It is important to address the many benefits that plastic provides in lieu of other concerns, e.g., plastic makes vehicles lighter, and therefore more fuel efficient.^[^ ^142^ ^]^ Plastic food wrapping also prolongs food shelf‐life and reduces excess food waste.^[^ ^143^ ^]^ Many plastic items have important functional benefits, their drawbacks should not be seen as arguments to remove all these applications from the market today; rather, they set the direction and focus for redesign, innovation, and behavior change.

However, plastics substantial overuse (especially for some single‐use items), is becoming an increasing environmental challenge. It is clear that our reliance and relationship with plastic as material needs to change but finding alternatives that provide the same qualities that plastic possess is not necessarily the answer, as alternative materials may have different environmental impacts. As an example, cotton clothing may become more popular as consumers try to find alternatives to plastic after awareness that a large majority of our clothes are made out of plastic. Although cotton is a natural material, it also has its own environmental problems. Cotton requires a substantial amount of water for its growth, depleting it from areas that might require it for other purposes.^[^ ^144^ ^]^ It is also more expensive than synthetic plastic clothing.

To find a solution to the growing amount of plastic waste, there have been developments in creating plastic formulations which state they deteriorate faster and/or have fewer impacts on the environment because their persistence is shorter.^[^ ^145^ ^]^ However, it was reported that biodegradable plastic formulations can persist in the soil and the marine environment for over 3 years. Compostable plastic tested in the same experiment completely disappeared in the marine environment within a 3 month period but remained intact in soil for 3 years. Therefore, it is not clear that such plastic formulations provide sufficiently advanced rates of deterioration to be advantageous in the context of reducing litter. Many plastics which claim to be biodegradable or compostable need to be disposed of to an industrial composter, with high heat and moisture, in order to deteriorate. Therefore, statements about the degradation of plastic products should be clearly linked to appropriate standards, made in conjunction with statements on the receiving environment for such degradation (e.g., commercial composter) and time scale to which those claims relate.

Hence, the potential negative repercussions of replacing conventional plastic alternative materials should be fully evaluated alongside any potential benefits. This process should consider: natural resource depletion, cost, accessibility, and consumer awareness. Industries are developing solutions aimed to stem the flow of or capture plastic getting into the environment. However, it is essential that any proposed solutions are fully tested for their efficiency and evaluated to understand their potential benefit.

### 7.6 Future Research

Lack of standardized protocols for plastic litter detection, sampling, and extraction creates issues for the comparability of data. Methods need to be improved in order to categorize different size classes (including nanoplastics), sampling procedures, analytical methods, and reference materials. Hartmann et al. recently proposed a definition and categorization framework. This framework went beyond size classes to include physicochemical properties (polymer composition, solid state, solubility) as defining criteria with size, shape, color, and origin as classifiers for categorization. The aim is that this framework could promote consensus building within the scientific and regulatory community based on a solid scientific foundation.^[^ ^36^ ^]^

There is also uncertainty about the specific extent and magnitude of the harm of plastic pollution in the marine environment. However, most agree that there is too much litter in the environment. The challenge is to take the most appropriate actions and to fit these interventions to particular causes of marine litter.

## 8 Conclusion

Solutions for addressing plastic pollution are available but will require coordinated action internationally and across a number of sectors/stakeholders. Governments and policy change have a pivotal role to play in creating the critical legislative framework to stimulate mitigation actions that contribute to a reduction in plastic waste at source, as well as encouraging cleaning up of plastic pollution on coastlines. Education and awareness of the problems produced by plastic debris are important as a step toward changing people's behavior with regard to plastic consumption, but knowledge alone is unlikely to be sufficient to change behavior.

In our view, this is an environmental problem that is largely avoidable. In short, the benefits of plastic can be realized without the need of end‐of‐life plastics to accumulate in the environment. Estimates of emissions to the environment indicate the severity of the issue. It is also clear that once in the environment, plastics are highly persistent and challenging to remove. The need for action is pressing and the scale of the problem indicates no single action will be sufficient. We need to simultaneously apply multiple actions including, reduction, re‐use, and recyclability as a matter of urgency.

## Conflict of Interest

The authors declare no conflict of interest.
